# Imaging retinal melanin: a review of current technologies

**DOI:** 10.1186/s13036-018-0124-5

**Published:** 2018-12-04

**Authors:** Maryse Lapierre-Landry, Joseph Carroll, Melissa C. Skala

**Affiliations:** 1 Morgridge Institute for Research, Madison, WI USA; 2 Department of Biomedical Engineering, Vanderbilt University, Nashville, TN USA; 3 Department of Cell Biology, Neurobiology & Anatomy, Medical College of Wisconsin, Milwaukee, WI USA; 4 Department of Ophthalmology & Visual Sciences, Medical College of Wisconsin, Milwaukee, WI USA; 5 Department of Biomedical Engineering, University of Wisconsin Madison, Madison, WI USA; 6 Department of Pediatrics, Case Western Reserve University, Cleveland, OH USA

**Keywords:** Retina, Retinal pigment epithelium, Choroid, Fundus reflectometry, Photoacoustic, Autofluorescence, Near-infrared, Optical coherence tomography, Photothermal, Polarization

## Abstract

The retinal pigment epithelium (RPE) is essential to the health of the retina and the proper functioning of the photoreceptors. The RPE is rich in melanosomes, which contain the pigment melanin. Changes in RPE pigmentation are seen with normal aging and in diseases such as albinism and age-related macular degeneration. However, most techniques used to this day to detect and quantify ocular melanin are performed ex vivo and are destructive to the tissue. There is a need for in vivo imaging of melanin both at the clinical and pre-clinical level to study how pigmentation changes can inform disease progression. In this manuscript, we review in vivo imaging techniques such as fundus photography, fundus reflectometry, near-infrared autofluorescence imaging, photoacoustic imaging, and functional optical coherence tomography that specifically detect melanin in the retina. These methods use different contrast mechanisms to detect melanin and provide images with different resolutions and field-of-views, making them complementary to each other.

## Background

Melanin is naturally present in the eye within the choroid, iris, and retinal pigment epithelium (RPE), a single layer of epithelial cells located posterior to the photoreceptors in the retina. The RPE plays an important role in the overall health of the retina, transporting nutrients from the blood vessels in the choriocapillaris to the photoreceptors, and disposing of retinal waste and metabolic end products [1]. An interruption in these functions can lead to degeneration of the retina, loss of the photoreceptors and eventually blindness. The melanin in the RPE is thought to play a protective role, absorbing excess light from the photoreceptors and protecting the retina from light-generated oxygen reactive species [2–4]. However, melanin in the RPE does not regenerate, and the damage accumulated over time from light exposure could affect the overall health of the RPE [2, 5]. In the past, most methods available to researchers to study melanin in the RPE were destructive to the tissue and labor intensive, which has led to a limited understanding of the role of melanin in the intact living eye. To further study the RPE, new imaging techniques have been developed to specifically detect and quantify melanin at the clinical and pre-clinical levels in patients and animal models.

Eye imaging has multiple roles, both to improve patient care and to perform basic research. Clinical imaging is used in patients to screen and diagnose eye conditions, plan and monitor ocular surgeries and evaluate treatment response [6, 7]. In animal models, non-invasive imaging methods enable observation of how different ocular structures interact with each other in a living system. Disease progression can be studied over time in the same animal, which can lead to the identification of new disease markers. Alternatively, new drugs can be dynamically evaluated, which could accelerate clinical translation. Fundus photography, scanning laser ophthalmoscopy (SLO) and optical coherence tomography (OCT) are all non-invasive imaging techniques that are part of the toolset for clinicians and researchers to image the eye. These techniques could be adapted to image melanin in the living eye and improve our knowledge of the RPE.

Changes in retinal pigmentation normally happen with aging [8] and are present in many ocular diseases. Albinism, for example, is characterized by various degrees of ocular hypopigmentation and is associated with low visual acuity and other visual abnormalities [2]. Retinitis pigmentosa, another example, is a group of genetic disorders that cause progressive visual loss and includes both photoreceptor degeneration and RPE cells loss [9]. Finally, age-related macular degeneration (AMD) is the most important cause of vision loss in adults above 65 years old in the US and involves dysfunction of the RPE and changes in pigmentation [10]. At early stages of the disease, AMD is usually characterized by changes in pigmentation and the presence of drusen. At later stages, “dry” AMD is characterized by regions of atrophy of the RPE and photoreceptors, while in “wet” AMD neovascular lesions invade the retina from the choroid and lead to vascular leakage, scaring and central vision loss [11]. In dry AMD, hyperpigmentation in the RPE (potentially from dysfunction in the RPE cells) followed by hypopigmentation (from the loss of RPE cells) could appear before dysfunction in the photoreceptors or choriocapillaris and could be predictive for the progression of the disease [11]. In wet AMD, it is possible that loss of the choriocapillaris causes the RPE cells to become hypoxic and to produce angiogenic substances, resulting in the formation of neovascular lesions [11]. To this day, there is no cure for AMD and vision loss cannot be reversed, although anti-VEGF treatment can slow down or stop disease progression [12–14].

Clinical imaging in the eye is already used to facilitate diagnosis, evaluate treatment response and reduce the need for repeated treatment in AMD [15, 16]. However, changes in pigmentations are still difficult to quantify since many non-invasive measurements are highly dependent on the optical properties of the eye and on the imaging parameters used. As a result, there are currently no standard in vivo techniques to quantify melanin levels in the eye.

The aim of this manuscript is to explore the different ways melanin can be imaged in the living eye. It is believed that light damage accumulated over time reduces the melanin’s ability to protect the retina. Imaging and quantifying melanin in the eye could provide information about the overall health of the RPE and of neighboring structures. As a result, melanin imaging could play a role in creating and evaluating new treatments in animal models or diagnosing ocular diseases before irreversible vision loss. The following key technologies enable non-invasive detection of melanin in the eye at the clinical and pre-clinical level and will be reviewed in this manuscript: fundus photography, fundus reflectometry, near-infrared autofluorescence imaging (NIR-AF), photoacoustic imaging (PA), optical coherence tomography (OCT), polarization-sensitive OCT (PS-OCT) and photothermal OCT (PT-OCT). A brief summary of existing ex vivo methods to quantify melanin in samples is also presented to provide context.

### Quantifying melanin ex vivo

Multiple methods have been developed to quantify melanin in cells or in ex vivo tissue samples. In early studies of the RPE, changes in pigmentation were observed qualitatively [17, 18] or quantitatively [19] by counting melanosomes on high resolution micrographs. To accelerate the process, melanin is now quantified using chemical degradation of the sample followed by high-performance liquid chromatography (HPLC) [20]. Electron spin resonance spectroscopy (ESR) has also been used to quantify melanin and characterize the different types of melanin pigments [5, 21, 22]. ESR spectroscopy measures the magnetic field strengths at which electrons in a sample can change their spin magnetic moment (from parallel to anti-parallel) by absorbing the energy from a microwave source of fixed frequency. The resulting spectrum of energy absorption as a function of magnetic field strength is specific to a given chemical compound and can be used to differentiate pigments. Melanin can also be quantified in terms of light absorption. Absorbance of solubilized melanin at a specific wavelength measured with a spectrophotometer is another technique used to quantify melanin in ex vivo samples [5, 23–25]. Light transmission measurements can also provide a measure of melanin concentration in tissue slices [26]. Ex vivo methods provide a highly specific and quantitative measurement of melanin and are used to study melanin production, distribution and degradation as a function of age and diseases. However, these methods cannot be used in live animal models to monitor diseases over time or test new treatments, and they cannot be translated to the clinic for use in patients. As such, in vivo techniques that can detect melanin have been a focus of many researchers.

### Fundus photography and fundus reflectometry

Fundus photography is a commonly used clinical imaging modality that produces a two-dimensional, *en face* color image of the retina where the optic nerve head, macula and major blood vessels can be seen. Most modern table-top fundus systems have a field-of-view of ~ 45° and do not require pupil dilation [27]. Fundus images can be recorded on 35 mm-film or with a digital camera [7]. The basic components of a fundus system are a white light source to illuminate the retina, a central obscuration in the illumination path (annular aperture), an objective lens to form an image using the reflected light from the retina, a zoom lens to correct for the patient’s refractive error, and a camera to detect the image [28]. This results in an annular illumination pattern at the pupil, a circular illumination pattern at the retina and a circular image detected at the camera. The annular illumination pattern at the pupil reduces the back reflection from the cornea and allow for a better detection of the reflected light from the retina. The illumination and collection paths can be combined with a beam splitter, or a mirror with a central hole to deflect the illumination path while transmitting the collected light [28].

Researchers and clinicians can visually assess changes in pigmentation based on the color of the retina as seen on fundus images. For example, multiple manual grading systems are used to evaluate fundus images in patients with AMD and the presence of hypopigmentation or hyperpigmentation is evaluated as part of the overall assessment [29]. Additionally, adaptive optics has been used to correct light aberrations in the eye, effectively improving the lateral resolution of fundus photography, and providing images of pigment migration over time in “dry” AMD [30]. However, this method of evaluating fundus images cannot differentiate between melanin contained in the RPE or the choroid, nor is it quantitative. To collect quantitative information from the fundus image, fundus reflectometry was developed.

Fundus reflectometry can be performed with a retinal densitometer, an instrument composed of a light source, some filters to change the wavelength of the light entering the eye and a detector such as a photomultiplier, capable of quantifying the light exiting the eye [31]. When performing fundus reflectometry using this technique, a high intensity white light is first sent to the eye to bleach the retina. A lower intensity light of a specific wavelength (e.g. 500 nm) is then sent to measure the presence of a pigment such as melanin [31, 32]. The light reflecting from the retina is then quantified as it is reaching the detector over time. In other instruments, a white light source is used to illuminate the retina and a spectrometer is used at the detector to measure the reflected light at multiple wavelengths [33]. Different theoretical models describing how incoming light would be reflected or absorbed by the different tissue layers of the retina can then be fitted to the recorded light, and properties such as the optical density of melanin can be calculated [34].

**Fig. 1:**
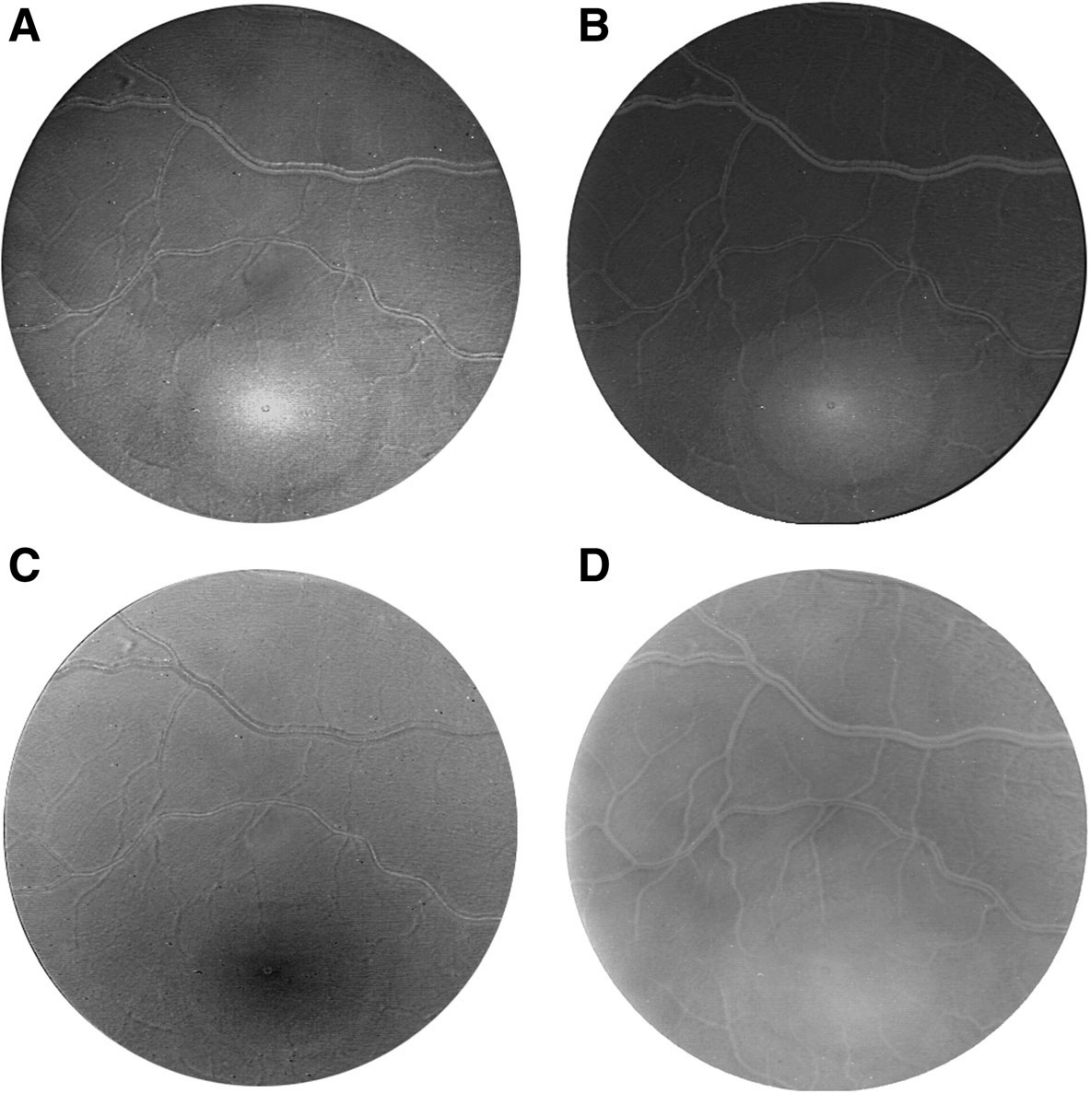
Pigment distribution obtained using four-wavelengths fundus reflectometry. Relative optical density at the fundus of (**a**) macular pigment obtained at 460 nm, (**b**) cone photopigment at 550 nm, (**c**) rod photopigment at 505 nm, and (**d**) melanin at 460 nm. Reprinted from [37] with permission from Elsevier

Fundus reflectometry studies have found different optical density values for choroidal melanin in healthy eyes based on different models [35, 36]. Recently, Hammer et al. used the adding-doubling approach, a technique used to simulate light distribution in a multi-layered tissue based on the reflection and transmission properties of a thin homogeneous tissue layer, to obtain relative concentrations of melanin in the RPE and choroid [33]. Bone et al. used a model based on the absorption of four components (macular pigments, cones and rods, and melanin) at four different wavelength to obtain 2D images of the fundus (see Fig.1) showing the relative optical density of melanin [37]. Kanis et al. compared the optical density of melanin from the right and left eye of patients and found a strong interocular correlation in healthy eyes [38]. This could open the door to diagnostic tests that evaluate large differences between melanin optical density between the eyes of a patient [38]. In another study by the same group, fundus reflectometry was used to image melanin in patients with age-related maculopathy (ARM) but did not detect differences in melanin optical density between healthy patients and patients with ARM, or between patients with different stages of ARM [32].

Fundus reflectometry is thus providing quantitative information about melanin distribution. This is an improvement over fundus photography where pigmentation changes can only be interpreted qualitatively. However, fundus reflectometry requires complex models to determine how the light entering the eye was scattered and absorbed by the different tissue layers of the eye. This can lead to widely varying results, including non-physical values of melanin optical density when layer thicknesses are not estimated correctly [33]. Additionally, while some models can produce 2D images of melanin distribution [37], most fundus reflectometry techniques do not produce an image, which renders data interpretation more difficult and does not account for heterogenous distributions of melanin. As a result, fundus reflectometry has not yet become a standard imaging technique in the clinic and has not been used extensively to study different diseases of the eye involving melanin. In conclusion, fundus reflectometry can obtain quantitative measurements of the melanin optical density, but the complex models required for quantification make this technology difficult to implement in practice.

### Near-infrared autofluorescence imaging (NIR-AF)

An alternative to fundus photography is scanning laser ophthalmoscopy (SLO) [39], which has enabled near-infrared autofluorescence imaging of the eye (NIR-AF). Like fundus photography, SLO produces two-dimensional *en face* images of the retina. However, a pinhole can be used to selectively collect light from a specific layer of the retina (~ 300 μm axial resolution [40]), which is not possible using a fundus camera [41]. Instead of a white light source, SLO uses a laser source focused onto a point and raster-scanned across the retina to build an image. This enables a small portion of the eye’s pupil to be used for illumination, while the rest of the pupil is used for light collection [41]. In comparison, fundus photography requires most of the pupil to be used for illumination (annular illumination pattern) with only the center of the pupil used for collection. As a result, SLO can be performed with illumination powers much lower than those required for fundus photography [39] and SLO is sensitive to lower levels of emitted light than fundus photography, enabling autofluorescence imaging of the eye [42]. Two endogenous fluorophores are most commonly imaged with SLO: lipofuscin and melanin [43, 44]. In most commercial and clinical SLO systems, the choice of excitation and emission wavelengths for fluorescence imaging is often dictated by the wavelengths used to image two exogenous fluorophores that are commonly used in the clinic to perform angiography: fluorescein and indocyanine green. However, these emission and excitation wavelengths are appropriate for lipofuscin (excitation: 488 nm, emission: > 500 nm, similar to fluorescein) and melanin imaging (excitation: 787 nm, emission: > 800 nm, similar to indocyanine green) [40, 45]. SLO thus enables qualitative imaging of the melanin and its distribution throughout the RPE.

The near-infrared autofluorescence signal of melanin in the retina was first reported, to our knowledge, by Piccolino et al. [46] in 1996 in a study that recorded near-infrared fluorescence before indocyanine green injection using fundus photography. At the time it was unclear what the source of the fluorescence signal was, and the authors hypothesized that it could be a combination of melanin, lipofuscin, and porphyrins. Later, Huang et al. confirmed that melanin in the skin and synthetic melanin produce fluorescence emission following near-infrared excitation [47]. Weinberger et al. confirmed the results from Piccolino et al. in the eye using an SLO system and further supported the hypothesis that the NIR fluorescence signal is caused by autofluorescence of melanin and not simply light reflected from the fundus (i.e. pseudofluorescence) [48]. Further evidence was provided by Keilhauer and Delori who imaged normal subjects and patients with AMD or other retinal diseases with NIR-AF, and determined that melanin in the RPE and choroid was a likely candidate for the source of the near-infrared autofluorescence signal [45]. Finally, Gibbs et al. demonstrated that the autofluorescence signal was specific to the melanosomes from the RPE and choroid by isolating them ex vivo [49].

**Fig. 2:**
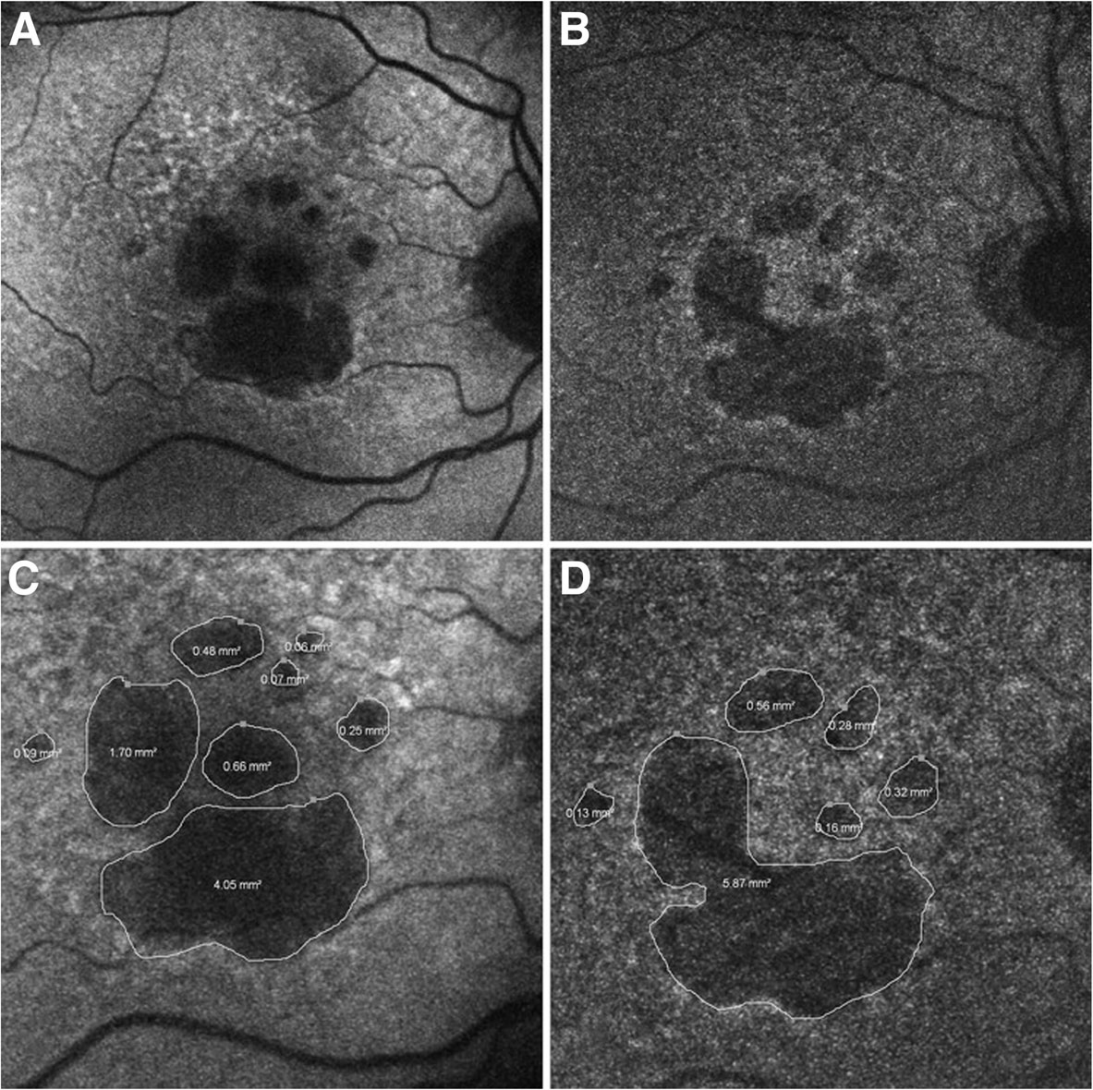
Geographic atrophy (GA) in the foveal region due to age-related macular degeneration (AMD) imaged with (**a**) short-wavelength autofluorescence (SW-AF) to detect lipofuscin, and (**b**) near-infrared autofluorescence (NIR-AF) to detect melanin. Areas of hypo-fluorescence (**c**, **d**) corresponds to GA. Larger areas of hypo-fluorescence are detected with (**c**) SW-AF compared to (**d**) NIR-AF, which may indicate that SW-AF overestimates areas affected by GA in the fovea. Reproduced from [50] with permission from BMJ Publishing Group Ltd.

NIR-AF was performed to detect melanin in patients and study diseases such as AMD [45, 48, 50–52] (see Fig.2), idiopathic choroidal neovascularization [53], chloroquine retinopathy [54], various inherited retinal diseases [55], *ABCA4*-associated retinal degenerations [56–58], retinitis pigmentosa [9, 59, 60], Usher syndromes [49, 61], Best vitelliform macular dystrophy [62], diabetic macular edema [63], central serous chorioretinopathy [64, 65], and torpedo maculopathy [66]. NIR-AF has multiple advantages as a melanin imaging technique: it offers a large imaging field-of-view, does not require exogenous contrast agents, is safe and comfortable for the patient, can be performed using commercially available equipment, and produces images that are easy to interpret by researchers and clinicians. However, NIR-AF does not have the axial resolution to produce three-dimensional images of the melanin distribution and it is likely that melanin from the RPE and choroid are both contributing to the NIR-AF signal. Additionally, the interpretation of the NIR-AF is mostly qualitative since the fluorescence intensity is highly dependent on imaging conditions. The NIR-AF signal can thus be quantified within one eye [45, 63] but it has been difficult to directly correlate the NIR-AF signal to an absolute measure of melanin concentration that would be valid across multiple eyes. However, quantitative autofluorescence has been performed in the eye to quantify lipofuscin in short-wavelength autofluorescence (SW-AF) images with the use of an internal fluorescent reference [67–69], which is encouraging for future quantitative autofluorescence measurements of melanin in the eye. In conclusion NIR-AF is easily performed using commercially available instruments and has been used to study multiple human diseases. However, RPE melanin cannot be separated from choroidal melanin and further research is needed to obtain quantitative NIR-AF results.

Fluorescence lifetime imaging ophthalmoscopy (FLIO) [70] is a technique similar to NIR-AF that not only measures the autofluorescence signal from fluorophores in the retina, but also the time it takes for fluorescence to be emitted following excitation (i.e. fluorescence lifetime). The fluorescence lifetime of a fluorophore such as melanin is highly dependent on the microenvironment but not dependent on fluorophore concentration, thus making FLIO particularly complementary to NIR-AF. The fluorescence lifetime of melanin has been recorded in hair samples [71]. However, the fluorescence lifetime signal obtained from the retina includes contributions not only from melanin but also from multiple fluorophores such as lipofuscin and macular pigments [70, 72, 73], and further studies are needed to isolate the lifetime signal of retinal melanin from other fluorophores in vivo.

### Photoacoustic imaging (PA)

Photoacoustic imaging (PA) is an ultrasound-based modality which can detect optical absorbers such as blood and melanin in the eye [74]. PA uses a pulsed-laser and an ultrasound transducer to detect absorbers in tissue. The laser light is absorbed by the contrast agent (e.g. melanin), which creates heat, rapid tissue expansion and an ultrasonic wave via the photoacoustic effect [75]. Such wave is detected by an ultrasound transducer coupled to the eye. Two types of information about the sample can then be obtained from the ultrasonic wave. First, a one-dimensional signal of absorption as a function of depth into the eye can be computed. The pulsed laser is then scanned across the sample to create two- or three-dimensional images of the absorbers within the sample. Second, the amplitude of the signal can be correlated to the absorption coefficient of the sample, and thus can serve as a measurement of the concentration of absorber (e.g. melanin) within the sample.

**Fig. 3:**
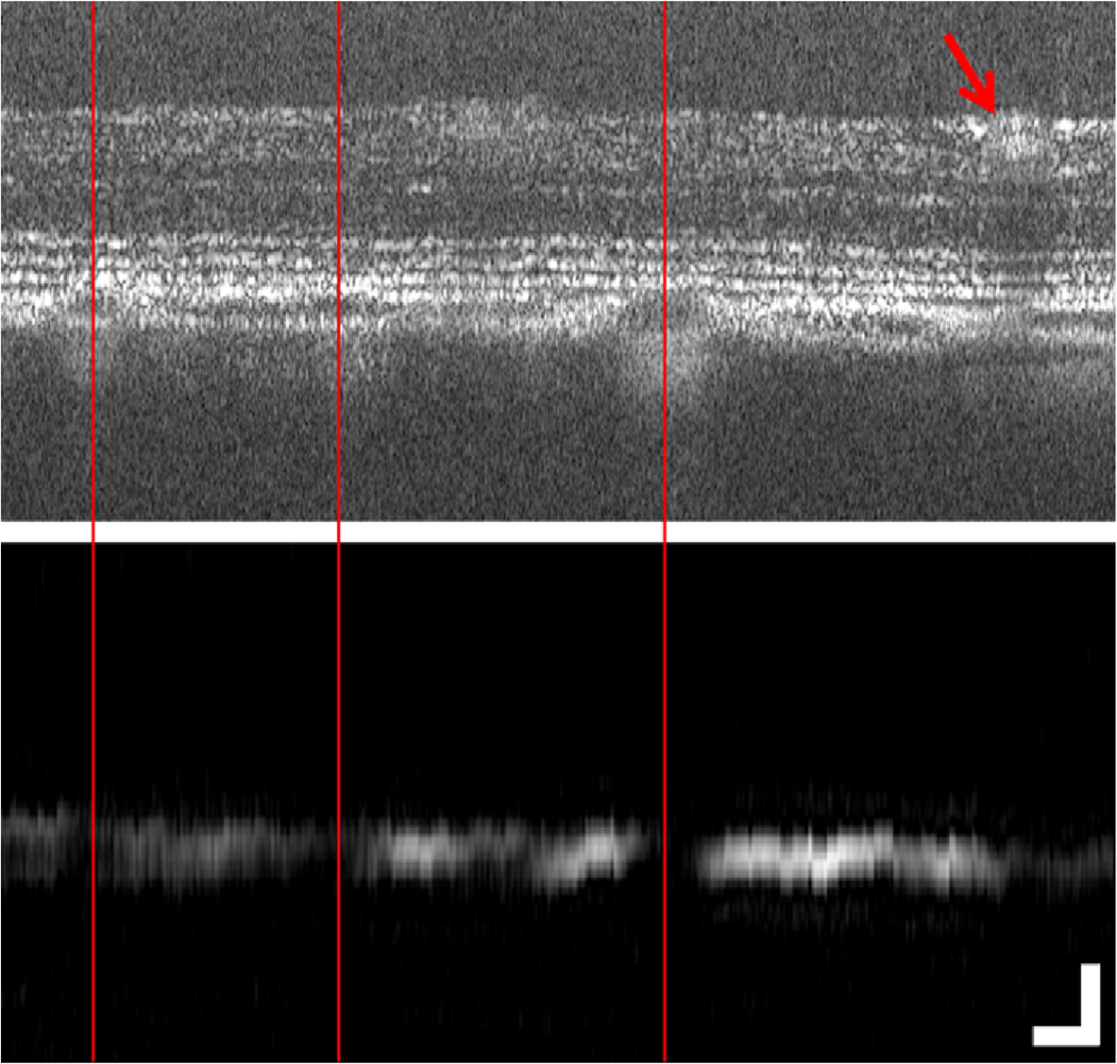
Optical coherence photoacoustic microscopy acquired in vivo in the rat eye. Top: OCT cross-sectional image showing the retinal tissue layers. Bottom: Co-registered photoacoustic image showing melanin in the RPE and choroid. Red arrow indicates retinal blood vessel. Scale bar: 100 μm. Reprinted from [84]. Copyright Optical Society of America

As a first demonstration, Silverman et al. acquired PA images of melanin in the iris in excised porcine eyes [76]. In the first in vivo demonstration, Jiao et al. integrated PA into an OCT system to collect photoacoustic images of the blood and melanin in the healthy rat retina with a 23 μm axial resolution [77]. This system used a needle transducer in contact with the eyelid to detect the ultrasound signal. Multiple follow-up studies have been produced by the same group. Zhang et al. added short-wavelength autofluorescence imaging to the PA system to detect lipofuscin in addition to melanin, first in retinal tissue [78], then in vivo in pigmented and albino rats [79]. Song et al. built upon this work and developed a multimodal system that includes PA, SLO, OCT and fluorescein angiography to image the eye [80]. The resulting system was able to simultaneously image tissue structure, retinal and choroidal blood vessels and melanin from the RPE and choroid in vivo in the retina of albino and pigmented rats [80]. This system was also adapted to image melanin in the mouse eye in Song et al. [81]. Previous PA systems by this group had used visible light (532 nm) to excite and detect ocular melanin, however, near-infrared light is less damaging to the eye than visible light. Liu et al. thus demonstrated in vivo melanin imaging in rats using a near-infrared laser (1064 nm) for PA excitation [82]. Liu et al. also combined a PA system to a fundus camera, which could visualize the position of the PA laser onto the retina and accelerate the alignment procedure when imaging melanin in rats [83]. Liu et al. were the first to perform in vivo optical coherence photoacoustic microscopy (PA and OCT combined using the same 800 nm wideband light source) in the rat eye, which lead to perfectly co-registered images of the tissue structure and melanin distribution (see Fig. 3) [84].

Images acquired up to this point had been qualitative and suffered from low axial resolution. PA has the potential to provide a quantitative reading of melanin concentration in the eye, similar to previous work imaging cutaneous melanin [85]. Shu et al. performed a Monte Carlo simulation to understand light absorption in the retina and evaluate the potential of PA imaging for quantitative imaging of melanin in the eye [86]. This model used blood absorption as a reference point for calibration. However, to specifically quantify RPE melanin and separate it from choroidal melanin, a higher axial resolution was necessary. Shu et al. used a micro-ring resonator detector to increase the axial resolution of their PA system (< 10 μm) and obtained images where the RPE and choroid can be distinguished in ex vivo porcine and human samples [87]. Quantitative melanin measurements of the choroid and RPE were then performed in ex vivo samples using a calibration curve obtained in phantoms.

PA imaging can provide volumetric images of ocular melanin, which was not possible using fundus reflectometry or NIR-AF fundus imaging. The increased axial resolution also allows for a more localized signal collection, and possibly for independent measurements of RPE and choroid melanin. PA imaging also relies on simpler light absorption and propagation models than fundus reflectometry, which may lead to more accurate measurements of melanin concentration. However, PA imaging has been demonstrated in few animal eye models and has yet to be demonstrated in the human eye. Additionally, no eye disease models have been explored using PA, thus it is unclear how the information provided by PA imaging will be used by eye researchers and clinicians in the future. In conclusion, PA imaging provides a quantitative measurement of melanin absorption and has the potential to separate signal from the RPE and the choroid. However, the technique has yet to be performed in the human eye.

### Optical coherence tomography (OCT)

OCT provides three-dimensional, high resolution images of the different tissue structures of the eye over a large field-of-view. First commercialized in 1996, OCT is now a standard imaging technique both for pre-clinical and clinical eye imaging [88–90]. OCT uses low-coherence interferometry to measure the echo time delay and intensity of the backscattered light as it penetrates tissue. Light is sent into a Michelson interferometer composed of a beam splitter, a sample arm (ending at the sample, in this case the retina) and a reference arm (ending with a reflective surface). A Fourier Transform of the resulting interferogram is used to obtain the OCT signal as a function of depth. The processed OCT signal is thus a complex signal where both the signal magnitude and phase vary as a function of depth. A single OCT scan (A-scan) is a one-dimensional measure of sample reflectivity as a function of depth. Two- and three-dimensional images can be acquired by raster-scanning the OCT beam over the sample. Typical OCT lateral resolution falls between 1.5 μm and 9 μm, dependent on the objective used and the imaging source wavelength. The axial resolution is determined by the imaging source wavelength and bandwidth, where, up to a point, small wavelengths and large bandwidth lead to better resolution. Ophthalmic OCT systems will often be centered around 850-860 nm with a 50 to 100 nm bandwidth, resulting in axial resolutions between 3 μm and 6 μm [91]. With such contrast mechanism and high axial resolution, different tissue layers such as the nerve fiber layer, photoreceptors, and RPE can be distinguished on OCT images [92].

Changes in melanin content are visualized as a change in RPE reflectivity on OCT images. Wilk et al. have analyzed these changes in OCT signal by comparing images obtained in wild-type and albino zebrafish, and by imaging patients with albinism [93]. Zhang et al. have also observed a change in intensity of the OCT signal in the RPE with dark adaptation in frogs [94]. However, the main source of contrast on OCT images is tissue backscattering, which provides limited functional information and low specificity when imaging melanin. Techniques such as polarization-sensitive and photothermal OCT have been developed to add functional contrast to OCT and can be used to specifically detect melanin.

**Fig. 4:**
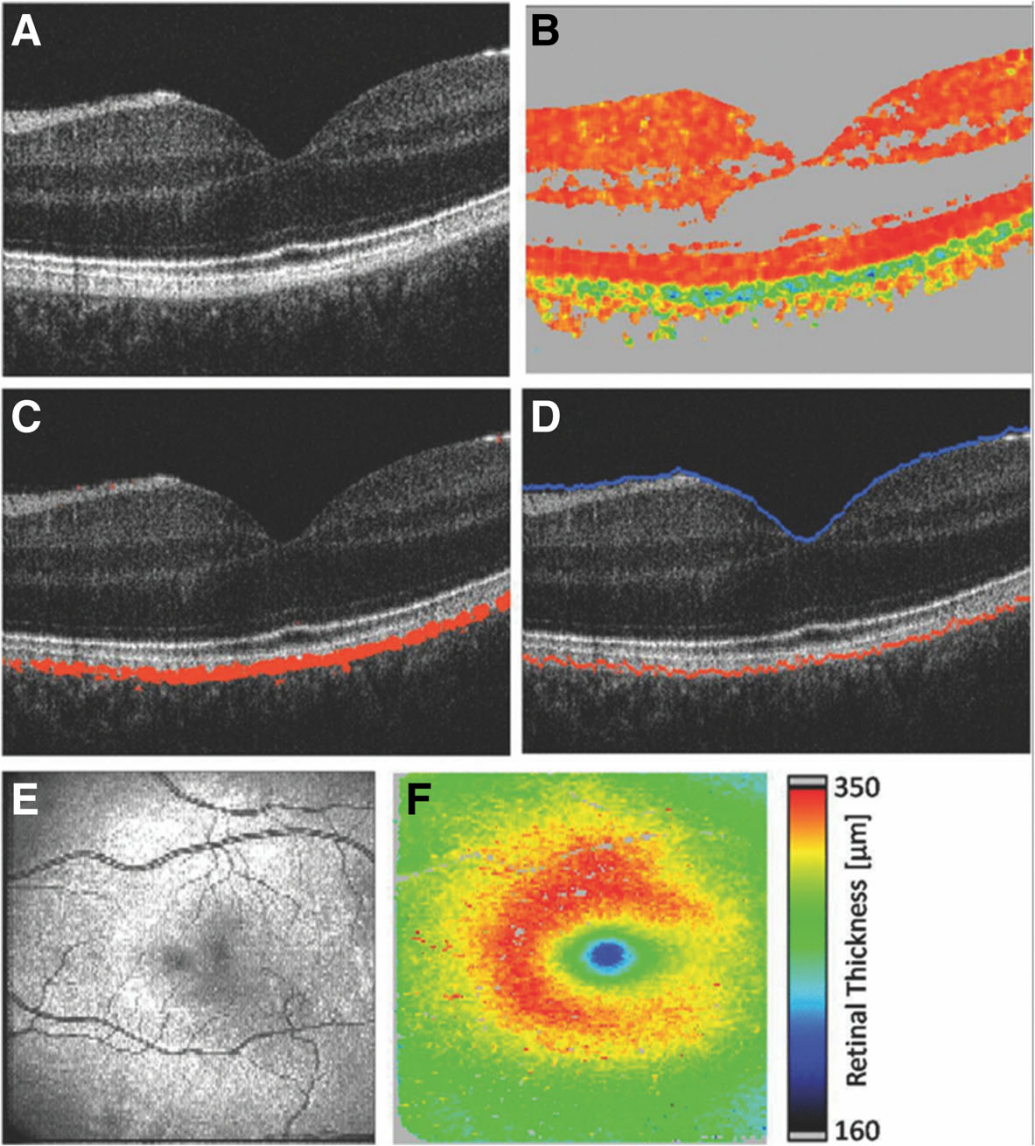
Segmenting the RPE and calculating retinal thicknesses using polarization-sensitive optical coherence tomography (PS-OCT): (**a**) OCT cross-sectional image of the retina, (**b**) degree of polarization uniformity (DOPU) image where the RPE has a low DOPU signal (green) compared to the rest of the retina, (**c**) Segmentation of the RPE based on low DOPU values, (**d**) position of the inner limiting membrane (blue) and RPE (red), (**e**) *en face* average intensity OCT image of the fundus, (**f**) corresponding retinal thickness calculated as the distance between the inner limiting membrane and the RPE. Reprinted from [109], under creative commons license

Polarization-sensitive OCT (PS-OCT) provides information about the birefringence of a sample and has been used to image the cornea and retina [95, 96]. To perform PS-OCT, incoming OCT light must be circularly polarized. After passing through the sample, the outgoing light then maintains an arbitrary ellipsoid polarization pattern determined by the composition of the sample [97]. From there, individual detectors are used to measure the vertical and horizontal components of the polarized light. Different algorithms are used to extract the polarizing properties of the sample, which can then be mapped onto a depth-resolved OCT intensity image. Pircher et al. first noted that light reflected from the RPE/Bruch’s membrane complex has a highly variable polarization when measured with PS-OCT in vivo in a volunteer [98]. Follow-up studies by different groups later confirmed that the polarization-scrambling layer was likely the RPE. This conclusion was made by comparing PS-OCT images obtained in healthy patients and images obtained in patients with RPE detachment, RPE tear, RPE atrophy, drusen or choroidal neovascular membrane [99–101]. Baumann et al. used melanin phantoms to determine the source of the PS-OCT signal within the RPE and observed that the degree of polarization uniformity (DOPU) is correlated with melanin concentration [102], a result later confirmed in rats [103]. However, this relationship was strongly dependent on the scattering properties of the sample, i.e. the size and shape of the melanin granules [102]. PS-OCT was also performed in pigmented rats and mice [104], albino rats [103–105], and patients with ocular albinism [102, 106], which confirmed the specificity of the PS-OCT signal to melanin. PS-OCT has been used to segment the RPE from 2D or 3D OCT data sets in healthy eyes [107] and in patients affected by AMD [108–111], RPE detachment [111] and pseudovitelliform dystrophies [108], and to compute retinal [109, 110] (see Fig. 4) or choroidal thickness [112]. Miura et al. showed that PS-OCT is complementary to other melanin imaging techniques by combining PS-OCT with polarization-sensitive SLO and NIR-AF to study RPE cells migration in patients with AMD [113]. PS-OCT has also been performed in combination with other functional OCT modalities, such as OCT angiography, to acquire information not only about the RPE but also about the structure and vasculature of eyes affected by AMD [111, 114, 115]. New algorithms [116] and instruments [117] have also been developed for PS-OCT to improve the detection of melanin and improve axial resolution down to < 1 μm.

**Fig. 5:**
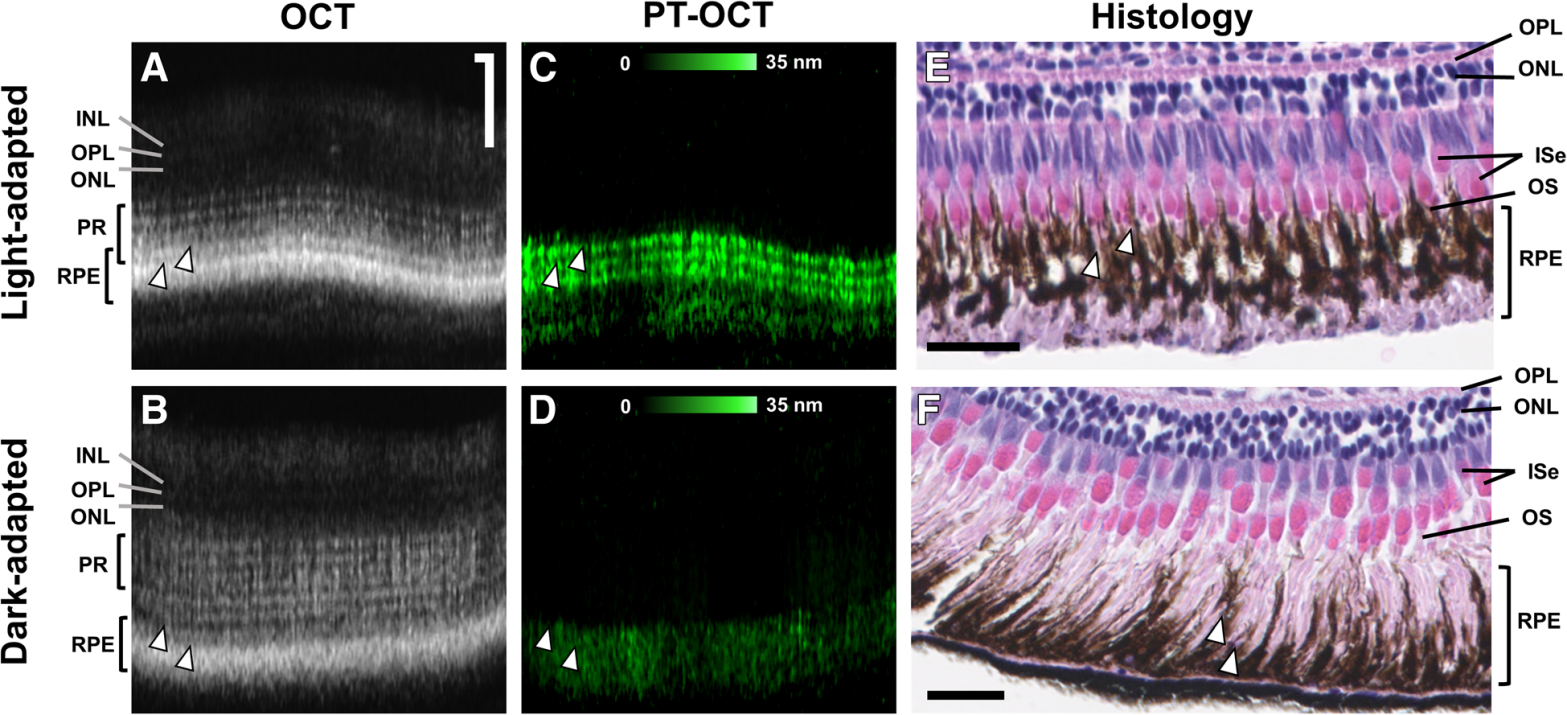
Melanosome migration in the zebrafish RPE due to light- and dark-adaptation as seen with photothermal optical coherence tomography (PT-OCT). **a**-**b** OCT cross-sectional images of the zebrafish retina with (**c**-**d**) co-registered PT-OCT images showing melanin distribution due to light- or dark-adaptation of the zebrafish, with (**e**-**f**) corresponding histology sections. White arrowheads indicate different structures where melanin is present [co-registered between images (**a**) and (**c**), and (**b**) and (**d**), approximate location for images (**e**) and (**f**)]. Scale bar: 50 μm for OCT and PT-OCT images, 25 μm for histology. INL, inner nuclear layer; OPL, outer plexiform layer; ONL, outer nuclear layer; PR, photoreceptors; ISe, photoreceptor inner segment ellipsoid zone; OS, photoreceptor outer segment. Reprinted from [122] under creative commons license

Photothermal OCT (PT-OCT) is another type of functional OCT technique [118, 119]. PT-OCT detects optical absorbers in tissues, with similar resolution and imaging depth as OCT. PT-OCT takes advantage of the photothermal effect, where photons absorbed by the contrast agent (e.g. melanin) are re-emitted as heat. To perform PT-OCT, an amplitude-modulated laser is combined to a phase-sensitive OCT system, with the wavelength of this additional laser corresponding to the absorption peak of the contrast agent. The increase in temperature following photon absorption causes a thermoelastic expansion surrounding the absorber, and a change in the refractive index of the tissue. Both phenomena cause a change in optical path length, which is detected as a change in the OCT phase signal. The PT-OCT signal intensity is proportional to the absorption coefficient of the tissue, which allows for quantitative measurements of the contrast agent concentration [119]. PT-OCT was first used to detect melanin by Makita et al. to image cutaneous melanin with PT-OCT [120]. PT-OCT was first performed in the eye by Lapierre-Landry et al. where signal from melanin was detected in the RPE in pigmented mice but absent in albino mice [121]. A follow-up study was performed in *tyrosinase*-mosaic zebrafish, a genetic line in which the zebrafish have pigmented and non-pigmented regions within the RPE of each eye. This study confirmed that the PT-OCT signal is specific to melanin in the zebrafish eye [122]. PT-OCT also detected melanosome migration within the RPE by comparing dark-adapted and light-adapted wild-type zebrafish (see Fig. 5) [122].

Both PS-OCT and PT-OCT are considered functional OCT techniques. They produce high-resolution images like OCT and they both can acquire volumetric images of the retina that are perfectly co-registered to the OCT intensity images. Both PS-OCT and PT-OCT instruments can be combined to other modalities such as OCT angiography to perform multimodal imaging. As PS-OCT and PT-OCT use different contrast mechanisms to detect melanin (polarization-scrambling and absorption, respectively), they can provide complementary information about melanin distribution within the retina. PS-OCT has the advantage of being low in illumination power, and it has been performed in both animal models and patients with a range of eye conditions. It has the potential of being a quantitative imaging modality for melanin, although it is unclear how the signal is dependent on the shape and size of the melanin granules and how small changes in pigmentations would be detected. PT-OCT has a more straightforward relationship with the absorption coefficient of a sample, with a linear increase in PT-OCT signal as a function of absorption. The PT-OCT signal is thus highly sensitive to small changes in pigmentation within the RPE. However, PT-OCT has yet to be performed in the human eye, and laser powers within safe levels (below ANSI standards) have only been demonstrated ex vivo [123]. In conclusion, both PS-OCT and PT-OCT have a high axial resolution and can separate the RPE from the choroid, but while PS-OCT has been used to study multiple diseases in both animal models and patients, PT-OCT has only been recently demonstrated in the eye in animal models.

## Conclusion

Melanin is present in the iris, choroid, and RPE, and may act as a protector to the photoreceptors to promote the overall health of the retina. Changes in pigmentation are observed in diseases such as albinism, retinitis pigmentosa and AMD, and studying these pigmentation changes could offer insights on disease mechanism, disease progression and treatment options. Here we reviewed non-invasive techniques to detect and quantify retinal melanin in the living eye. These methods have advantages over traditionally used ex vivo methods, since they can be used for longitudinal studies in animal models, where cost, time, labor and inter-animal variability are reduced by imaging the same animal over many time points. Many non-invasive imaging methods can also be used in patients for diagnosis and treatment, which is not possible with ex vivo methods.

In this review, we covered multiple techniques that have been used to detect melanin using a variety of contrast mechanisms. Changes in pigmentation can be seen using fundus photography, but observations are only qualitative and the signal produced by melanin contained in the RPE cannot be separated from the signal produced in the choroid. Fundus reflectometry can quantify melanin in the RPE, but the complex models required for quantification make this technology difficult to implement in practice. NIR-AF can be accomplished using commercially available SLO instruments and produces images that are simple to interpret by a clinician. However, it is difficult to quantify melanin across multiple eyes using NIR-AF and RPE melanin cannot be separated from choroidal melanin with the existing axial sectioning capabilities of commercial SLOs. PA imaging uses an ultrasound transducer to produce three-dimensional images of the eye and a pulsed laser to detect optical absorbers such as melanin. The PA signal intensity is directly correlated with melanin absorption and recent advances have made it possible to separate the signal from RPE and the choroid. However, the axial resolution is still limited, and the technique has not been performed the human eye. Finally, OCT is a three-dimensional imaging technique that is commonly used in the clinic. Since melanin does not produce a specific change in OCT signal, functional OCT techniques such as PS-OCT and PT-OCT have been developed to detect melanin using its polarization-scrambling properties and its absorption properties, respectively. While PS-OCT has been used in multiple animal models and in patients, PT-OCT is an emerging technology that has only been recently demonstrated in the eye.

These methods are complementary to each other and together provide researchers and clinicians with a range of field-of-views, in 2D or 3D, obtained at different resolutions, and using properties such as absorption, fluorescence or light polarization as contrast mechanisms. We expect that in the future, in vivo experiments will lead to a better understanding of the role of melanin in the retina, which could lead to new diagnosis methods and new treatment options.

## Abbreviations

AMD: Age-related macular degeneration
ARM: Age-related maculopathy
DOPU: Degree of polarization uniformity
ESR: Electron spin resonance
FLIO: Fluorescence lifetime imaging ophthalmoscopy
GA: Geographic atrophy
HPLC: High-performance liquid chromatography
NIR-AF: Near-infrared autofluorescence
OCT: Optical coherence tomography
PA: Photoacoustic
PS-OCT: Polarization-sensitive optical coherence tomography
PT-OCT: Photothermal optical coherence tomography
RPE: Retinal pigment epithelium
SLO: Scanning laser ophthalmoscopy
SW-AF: Short-wavelength autofluorescence
